# Dealing with Too Little: The Direct Experience of Scarcity does not Affect Snack Intake

**DOI:** 10.1111/aphw.12163

**Published:** 2019-04-09

**Authors:** Sofie van Rongen, Kirsten Verkooijen, Emely de Vet

**Affiliations:** ^1^ Wageningen University & Research The Netherlands

**Keywords:** calorie consumption, diet quality, eating behaviour, low income, scarcity, trade‐off making

## Abstract

**Background:**

The experience of scarcity provides an explanation for the relatively unhealthy diets of people with low income. Causal evidence for an effect of direct experiences of scarcity on eating behaviour is lacking.

**Methods:**

Two studies (*N *=* *81, *N *=* *115) tested and refined a self‐developed trade‐off task, in which participants' resources were restricted (scarcity condition) or unrestricted (no‐scarcity condition), for manipulating experiences of scarcity. Two further studies (*N *=* *95, *N *=* *122) were performed to test whether scarcity results in greater calorie consumption from snacks and lower self‐reported self‐regulation of eating.

**Results:**

The scarcity manipulation appeared successful. A significant main effect of scarcity on eating was not found; however, an interaction effect between hunger and scarcity bordered on significance, such that those in the scarcity condition consumed more calories under low hunger. In the second experiment, participants were instructed to eat prior to participation to lower their hunger level. No difference between conditions was found in calorie consumption and self‐regulation of eating.

**Conclusion:**

Although the trade‐off task appeared to evoke scarcity experiences, the present research could not support the notion that these result in unhealthier eating. A more nuanced view of the influence of scarcity on eating is needed.

## INTRODUCTION

Diet‐related diseases such as obesity, diabetes, and heart disease are approaching epidemic levels in many parts of the world (Deitel, 2003; Fardet & Boirie, 2014; Wagner & Brath, 2012). It has been well established that diet quality differs over income groups: people with low income have more unhealthy diets (Drewnowski & Specter, 2004; Ricciuto & Tarasuk, 2007). Moreover, lower incomes are associated with higher obesity rates (Schoenborn, Adams, & Barnes, 2002) in both developed and developing countries (James, Leach, Kalamara, & Shayeghi, 2001). A dominant explanation is that a low income induces a selection of less expensive unhealthy, high energy‐dense foods. However, research focused on the role of food prices and perceived affordability of healthy foods in diet quality of different income groups has shown inconsistent findings (e.g. Dijkstra et al., 2018; Lee, Kane, Ramsey, Good, & Dick, 2016). Hence, there may be other explanations for why having a low income contributes to unhealthy eating. This research focuses on a more fundamental reason for this relationship: psychological consequences of the experience of scarcity resulting from a low income.

Relatively recently, a psychological perspective of financial scarcity has been put forward that provides an underlying explanation for anomalies in a wide variety of behaviours, including healthy eating. This approach, also labelled “scarcity theory” (Mullainathan & Shafir, 2013; Shah, Mullainathan, & Shafir, 2012), primarily advocates that the experience of scarcity (i.e. “a subjective sense of having more needs than resources”; Mullainathan & Shafir, 2014, p. 86) negatively affects cognitive capacity, which subsequently results in behaviours that are in contrast to one's long‐term interest. Although the adverse impact of scarcity on eating behaviour as an explanation for unhealthy diets among people with low income has been suggested before (Mullainathan & Shafir, 2013; Spears, 2011), empirical evidence remains scarce. A recent cross‐sectional study showed that financial scarcity (financial strain) indeed related negatively to self‐reported health behaviours including fruit and vegetable intake (Beenackers, Oude Groeniger, van Lenthe, & Kamphuis, 2017), whereas a longitudinal study found that financial strain had limited to no effect on diet‐related health outcomes including being overweight (Prentice, McKillop, & French, 2017). To our knowledge, Bratanova, Loughnan, Klein, Claassen, and Wood (2016) showed the first experimental evidence for a causal effect of perceptions of poverty on unhealthy eating. They found that students writing about their own experiences with poverty (versus wealth) consumed more calories from snacks. The present study is more in line with scarcity theory and aims to expand on these first results by experimentally testing the impact of direct experiences of scarcity on snack consumption rather than by reliving or imagining situations of poverty.

### Scarcity Theory and its Relation to Unhealthy Eating

Essentially, the *perception* of scarcity of resources is the feeling that one has more needs than resources, or in other words, that one's resources are *too* little for the available options that would satisfy one's needs or desires. Having insufficient resources then forces daily difficult decision‐making involving trade‐offs and sacrifices, thereby enhancing the sense of having too little (Mullainathan & Shafir, 2013). As illustrated by Mullainathan and Shafir (2013), one could compare a situation of scarcity and trade‐off making with holiday packing with a small suitcase (representing a small budget); one has to think hard what to include and what could be left out. Fundamental to scarcity theory is that this experience of scarcity captures our attention: people tend to automatically focus on immediate problems and urgent unmet needs. Since people are limited in their attention and cognitive processing capacity (e.g. Kahneman, 1973), a preoccupation with immediate unmet needs and difficult trade‐offs reduces the cognitive capacity available for other (future) responsibilities (Mullainathan & Shafir, 2013). Cognitive capacity may deteriorate even further by the stress and negative affect associated with scarcity, which can further increase impulsiveness (Haushofer & Fehr, 2014). Notably, direct evidence for the negative effect of scarcity on cognitive capacity and control is scarce. Two revealing experimental studies showed that participants who were preoccupied with difficult (versus easy) hypothetical financial decisions (Mani, Mullainathan, Shafir, & Zhao, 2013) or who received few (versus many) guesses in a word puzzle (Shah et al., 2012) performed worse on a computerised cognitive control task (spatial incompatibility task; see also Davidson, Amso, Anderson, & Diamond, 2006). Furthermore, Spears (2011) revealed that participants who received a smaller (versus larger) choice “budget” to choose from free gifts, executed less self‐control as indicated by the duration of squeezing a handgrip and performance on a numerical Stroop task.

Notably, scarcity theory harmonises with self‐regulation theory, which is concerned with immediate urges on one hand and long‐term goals (e.g. health) on the other (Baumeister & Vohs, 2007). Also, the ability to self‐regulate is assumed to be limited and subject to situational circumstances including cognitive load, stress, and affect (Hofmann, Friese, & Wiers, 2008), all of which have been related to the experience of scarcity (e.g. Haushofer & Fehr, 2014; Shah et al., 2012). Applied to eating behaviour this means that when cognitive capacity to act in line with health goals is (temporarily) diminished, the influence of tempting food stimuli on behaviour is enhanced (Hofmann, Rauch, & Gawronski, 2007). Indeed, experimental studies have shown that unhealthy eating behaviours can result from situationally decreased cognitive capacity. For example, applying a commonly used manipulation for cognitive load, namely asking participants to remember a seven‐digit (versus a three‐digit) number, experimental studies have shown that this cognitive load increased unhealthy food choices (Shiv & Fedorikhin, 1999) and calorie consumption (Ward & Mann, 2000). Based on these insights, it has been reasoned that experiencing scarcity, resembling cognitive load, increases susceptibility to consume tempting foods (Mullainathan & Shafir, 2013; Spears, 2011). Apart from the idea that scarcity experiences lead to less cognitive capacity and self‐regulation, it is plausible that unhealthy eating may be a more direct result from a more present‐time focus stemming from the threatening nature of scarcity perceptions. Obtaining caloric resources in times of scarcity may reflect an adaptive motivation to compensate for (future) scarcity of resources (see also Laran & Salerno, 2013; Swaffield & Roberts, 2015). Overall, the present research may provide additional support for the notion that food consumption in response to scarcity is not domain restricted but may also be evoked by non‐food resources (Briers & Laporte, 2013; Koles, Wells, & Tadajewski, 2018). Especially when people with low income live in neighbourhoods in which they are more frequently exposed to unhealthy (often tempting) foods (Cummins, 2003; Darmon & Drewnowski, 2008), adopting a healthy diet may be a difficult endeavour when also experiencing scarcity.

### The Present Research

Our aim was to experimentally investigate whether direct experiences of scarcity indeed result in unhealthier eating in terms of calorie intake from snacks. Specifically, by restricting the amount of choice resources in a trade‐off task (based on Spears, 2011), we aimed to evoke real‐time experiences of scarcity including a sense of having too little and trade‐off making, so as to mimic daily difficult decision‐making with limited resources. Four experimental studies with independent student samples were performed. We designed the task such that the selection of options (goods and services) served to achieve a salient, concrete goal which was to organise a successful student party. In accordance with scarcity theory, we argued that experiences of scarcity can be induced as long as available resources to select options are insufficient to satisfy goal‐related needs and desires. In Studies 1 and 3, we tested the feasibility and the refinement (respectively) of the trade‐off task directed at organising a successful party for manipulating experiences of scarcity. Notably, in the limited number of studies on the cognitive effects of scarcity of resources it was not checked whether objectively receiving few versus many resources indeed resulted in different experiences of scarcity. Checking the validity of the manipulation was considered particularly important because although the experience of scarcity is socially contextualised, it also depends on the subjective evaluation (own tastes) to what extent needs and desires are met (Mullainathan & Shafir, 2013). In Studies 2 and 4, we tested the effect of scarcity on calorie consumption and self‐reported indicators of self‐regulation of eating in an experimental laboratory setting. Participants were requested to taste high‐caloric snacks while performing the trade‐off task. Eating large amounts of snacks which are usually considered tasty (provide immediate pleasure) but unhealthy (have a long‐term consideration) is generally seen as a self‐defeating behaviour, especially if people have the goal to act otherwise (see also Brownell, 1991; Heatherton, Polivy, & Herman, 1991).

## STUDY 1: TESTING A MANIPULATION OF SCARCITY

In Study 1 we tested the feasibility of a trade‐off task for manipulating experiences of scarcity. The trade‐off task was inspired by a study of Spears (2011) in which participants could either choose two gifts (“rich” condition) or one gift (“poor” condition) out of three gifts. Likewise, our manipulation aimed to involve difficult decision‐making processes imposed by a scarcity of choice resources on one hand and the availability of desirable options on the other hand.

### Method

#### Participants, Design, and Procedure

A total of 81 undergraduate students (22 men) with a mean age of 21.27 (*SD *=* *1.73, range 19–28) voluntarily completed a trade‐off task during a course lecture. Participants received a hypothetical scenario on paper that described that the participant was given the unique responsibility to organise, on behalf of the university, a successful party for fellow students. All participants were presented with a list of nine categories of goods and services desirable for a party (e.g. “drinks”, “promotion”). Each category consisted of three equally attractive alternative choice options.^1^ For instance, in the category “drinks”, the three options listed were beer, wine, and soda, and in the category “promotion” were the options email, social media, and posters/flyers (see Supplemental Information for the entire trade‐off task). Participants in one half of the lecture room were assigned to a scarcity (experimental) condition where participants were only allowed to choose one option per category. The other half of participants were assigned to the no‐scarcity condition (control) where multiple (up to three) options per category could be selected. After the trade‐off task, participants completed a questionnaire (self‐report instrument, see below) to assess direct scarcity perceptions and potential experiences of decision‐making under scarcity. For explorative purposes, psychological states suggested to result from scarcity were measured on 7‐point scales, including mental engagement, cognitive load, and affect (see Supplemental Information for more details).

#### Measures

##### Self‐report instrument: Scarcity and decision‐making experiences

Items were based on specific definitions of scarcity perceptions as described by Mullainathan and Shafir (2013). Specifically, five items pertained to the experience of having more needs than resources and four items pertained to having to make trade‐offs and sacrifices. Additionally, four items pertained to experiences potentially involved in decision‐making under scarcity, including freedom of choice, choice overload, indecisiveness, and uncertainty. The answer scale ranged from 1 (*strongly disagree*) to 7 (*strongly agree*). To validate the different dimensions in the self‐report instrument, a principal component analysis was conducted with orthogonal rotation (varimax). Examination of the scree plot and eigenvalues over 1 suggested the presence of three components, in combination accounting for 73.71 per cent of the variance. Based on saliently loading items of the three components (all loadings > 0.59), three reliable subscales (.71 < Cronbach's α < .94) were constructed, which we labelled “making‐trade‐offs”, “need for more”, and “indecisiveness”, respectively. Mean scores on these scales were computed. One item, concerning freedom of choice, did not load high on any of the components (loadings < 0.4) and was therefore removed from the total set of items. See Table 1 for the 12 items included, their factor loadings for the three components, and the corresponding scales.

**Table 1:** Items and their Factor Loadings for the Three Scales and Results of *t*‐tests Comparing the Two Conditions on Experienced Scarcity (“Making trade‐offs” and “Need for more”) and Experiences Related to Decision‐Making under Scarcity (“Indecisiveness”) in Study 1

Scales and items^a^^*,*^ b	Factor loadings	Scarcity condition (N = 41) Mean (SD)	No‐scarcity condition (N = 40) Mean (SD)	t‐test (df = 79)	Cohen's d	95% CI	
						Lower Bound	Upper Bound
Making trade‐offs		5.42 (0.90)	2.53 (1.35)	11.29^d^	2.51	2.38	3.40
I needed to give up other choices	0.856						
I had to make a trade‐off to come to a choice	0.876						
I had difficulty choosing	0.823						
Making a choice meant not having another attractive option	0.859						
Need for more		5.36 (0.89)	3.86 (1.74)	4.81^d^	1.09	0.89	2.10
I wanted to choose more than I could	0.827						
I could choose too little	0.842						
I had enough choice^c^	0.837						
I wanted to be able to choose more	0.831						
I was restricted in my choice	0.762						
Indecisiveness		3.36 (0.93)	2.37 (1.24)	4.05^d^	0.90	0.50	1.48
I was overwhelmed with choices	0.799						
I was indecisive	0.730						
I was uncertain	0.590						

*Note*

a Questionnaire started with the phrase “During choosing I had the feeling that…”.

b Answered on scale ranging from 1 to 7.

c Responses recoded.

d *p *<* *.001.

### Results

Independent *t*‐tests showed that participants in the scarcity condition scored significantly higher than participants in the no‐scarcity condition on the three scales. Table 1 reports the mean scores and standard deviations (*SD*s) per condition on each scale, and the corresponding test‐statistics, Cohen's *d* effect sizes, and confidence intervals. See Supplemental Table 1 for results of states related to scarcity, which shows that the scarcity manipulation had an effect on engagement (*p *=* *.02) and cognitive load (*p *=* *.02), but not on affect (*p *=* *.35).

### Discussion

The manipulation was considered successful as participants in the scarcity condition indicated more scarcity experiences (in terms of a need for more and trade‐off making) compared to participants in the no‐scarcity condition.

## STUDY 2: TESTING THE EFFECT OF SCARCITY ON UNHEALTHY FOOD INTAKE

In Study 2 we examined the impact of scarcity on unhealthy food intake. While completing the trade‐off task as developed in Study 1, participants were requested to taste high‐caloric snacks. The trade‐off task and the “tasting task” were performed simultaneously to be consistent with scarcity theory. Mullainathan and Shafir (2013) suggest that scarcity and the preoccupation it causes has an immediate effect, resembling cognitive load. It was hypothesised that participants in the scarcity condition consumed more calories from snacks than participants in the no‐scarcity condition. Furthermore, following the reasoning that scarcity reduces cognitive capacity and thereby undermines self‐regulation of eating in the presence of tempting snacks (immediately pleasurable and high calorie foods), we also examined whether the scarcity condition reported a higher desire for food and lower inhibition of eating (Hofmann et al., 2008; Strack & Deutsch, 2004).

### Method

#### Participants and Design

Students were recruited via email, social media, flyers, posters, direct person‐to‐person, and during course lectures. G*power was used to calculate the sample size needed to detect an effect size of f = 0.30, which was sourced from two previous studies that experimentally tested the effect of poverty/scarcity on calorie intake from snacks (Bratanova et al., 2016; Laran & Salerno, 2013). To reach at least a power of 80 per cent (alpha of 0.05), a total sample size of *N* = 90 was required for data analysis. We did not use a predefined stopping rule. Instead, experiments were continued for the full 3 weeks during which the laboratory rooms were available, eventually resulting in a laboratory visit by 104 students. After pre‐testing the procedure among three students, the experimental protocol was finalised. A total of 101 students participated in a two‐group between‐subjects experiment in exchange for a small monetary reward of 5 euros. We excluded six participants from analysis: three participants had a food allergy related to the presented snacks, and three participants did not adhere to instructions (two participants did not eat any snacks and one participant grabbed a handful of snacks after the experiment had finished). As a result, the sample for analysis consisted of 95 participants (12 men) with an average age of 20.83 (*SD *=* *2.20, range 18–28).

#### Manipulation

The scarcity manipulation involved the trade‐off task as explained in Study 1. Based on the frequency distribution of chosen options in Study 1, small adaptations to the trade‐off task were made. One category with two infrequently options chosen was removed from the task, and two other infrequently chosen options were replaced by other, intuitively more attractive options (see Supplemental Information for the adapted trade‐off task).

#### Procedure and Measures

Participants who signed up for the study were scheduled for an individual test session during daytimes (between 09.30 and 17.00 h). Participants were randomly assigned to either the scarcity (experimental) or no‐scarcity (control) condition using a computer‐generated numbers list. Upon entering the laboratory, participants read and signed the informed consent. Thereafter, the first questionnaire was administered which included demographic measures (i.e. age, gender, year and field of study), an item measuring hunger (“How hungry are you at this moment?”, embedded among four filler state items, i.e. thirst, stress, mood, and fatigue), and items measuring healthy eating goal and restraint eating goal (“In daily life I try to eat healthily” and “In daily life I try not to eat too much”, embedded among eight filler daily life goal items, e.g. physical activity, relaxation). All items of this first questionnaire were answered on a 7‐point scale ranging from 1 (*not at all*) to 7 (*very much*). In the adjacent room (decorated with party items), participants were seated at the table with the trade‐off task sheets, a cup of water, and four bowls with different snacks (M&Ms, popcorn, crispy coated peanuts, and crisps). The four types of food were used to balance for a preference for sweet or savoury snacks. These snacks were deemed to be tasty and unhealthy foods (all contained a minimum energy value of 400 Kcal per 100 g) and consumption thereof is likely susceptible to self‐regulation resources. All bowls (12 cm diameter and 8 cm deep) were fully filled so that an individual could eat substantial amounts without creating any obvious indication of consumption (target weights were crisps 80 g, crispy coated peanuts 230 g, popcorn 70 g, and M&Ms 400 g). As a cover story, participants were told that we investigated students' views on the ideal student party, and that the party decoration served to appeal to one's imagination in the task, as well as to explore the influence of party atmosphere on taste perception. Participants were told to consume whatever and as much of the snack as they desired during the task. After 8 minutes, which was considered sufficient time to complete the task and taste the snacks, the experimenter returned, placed the snacks at the far end of the room, and presented the participants with the last questionnaire that included the scarcity and decision‐making experiences questionnaire, scarcity‐related states during the task (stress was measured in addition), boredom after the task (“How bored were you after completing the task?”), desire for snacks (“How much did you want [snack]?”), liking of consumed snacks (e.g. “The crisps were tasty”), and inhibition of eating (“Did you inhibit yourself from consuming snacks?”) (in that order). The 7‐point answer scale of the items of the last questionnaire ranged from *strongly disagree* to *strongly agree* for items formulated as statements and from *not at all* to *very much* for items formulated as questions. A final question asked participants to state what they thought the purpose of this study was. None of the participants mentioned the true purpose of the study (i.e. the relation between scarcity and snack intake).^2^ Finally, participants were thanked, reimbursed, and debriefed upon request by email. Each of the bowls of snacks was unobtrusively weighed with a kitchen scale (0.1 g precision) before and after participation, and these eight weight values were all collected in a predesigned table on a sheet of paper coded with the participant number. Consumed calories per snack were calculated based on the consumed weight and the energy content indicated on the product label. A composite score was formed, summing together the consumed calories of the four snacks.

### Results

#### Descriptives and Comparability between Conditions

As calorie consumption was highly positively skewed, a logarithmic transformation was used to normalise the distribution of residuals. Participants on average indicated having a goal to eat healthily (*M *=* *5.49, *SD *=* *1.01), and a somewhat restraint eating goal (*M *=* *4.40, *SD *=* *1.28). Participants liked the snacks they consumed, with the M&Ms gaining the highest rating, which indicates that the snacks were indeed tasty and pleasurable to consume (*M*_M&Ms_ = 5.99, *SD *=* *1.02; *M*_popcorn_ = 4.89, *SD *=* *1.76; *M*_crisp*s*_ = 5.25, *SD *=* *1.49; *M*_crispy coated peanuts_ = 4.46, *SD *=* *1.83). Conditions did not differ on pre‐test variables age, hunger, healthy eating goal, restraint goal, *t*(93) > 0.25, *p *> .18, and gender, *χ* ^*2*^(1, *N* = 95) = 1.25, *p *=* *.26, suggesting that our randomisation was successful. Neither did conditions differ on the post‐test variable boredom, *t*(93) = 0.19, *p *=* *.85, indicating that we can rule out this potential alternative explanation for consumption. An analysis of significant correlations between the control variables and dependent variables resulted in the identification of gender and hunger as relevant covariates. Means, *SD*s and correlations of the variables under study are reported in Supplemental Table 2.

#### Manipulation Check and Exploration of States

Independent *t*‐tests revealed that participants in the scarcity condition scored significantly higher than participants in the no‐scarcity condition on the experienced scarcity scales “need for more” and “making trade‐offs”, as well as on “indecisiveness”. Hence, the manipulation appears successful. Table 2 reports the mean scores and *SD*s per condition on each of these scales, and the corresponding test‐statistics, Cohen's *d* effect sizes, and confidence intervals.

**Table 2:** Results of *t*‐tests (Studies 2 and 4) and Post‐Hoc Dunnett's Test (Study 3) Comparing the Conditions on Experienced Scarcity (“Need for More” and “Making Trade‐offs”) and “Indecisiveness”

Scale (Cronbach's α)	Conditions		t‐test	Cohen's d	95% CI	
	Scarcity Mean (SD)	No‐scarcity No‐scarcity extra Mean (SD)			Lower bound	Upper bound
Study 2	*N *=* *49	*N *=* *46	*df *=* *93			
Need for more (0.87)	5.41 (0.86)	4.05 (1.50)	5.39***	1.11	0.87	1.86
Making trade‐offs (0.91)	5.62 (0.90)	2.73 (1.16)	13.57***	2.78	2.46	3.31
Indecisiveness (0.68)	2.80 (1.09)	2.17 (0.89)	3.01**	0.63	0.22	1.04
Study 3	*N *=* *39	*N *=* *38 *N *=* *38				
Need for more (0.88)	5.53 (0.84)^a^	3.36 (1.51)^b^	N/A	1.78	−2.79	−1.55
		*2.98 (1.18)^b^*	N/A	2.50	−3.17	−1.94
Making trade‐offs (0.91)	5.60 (1.00)^a^	2.53 (1.28)^b^	N/A	2.67	−3.66	−2.50
		*2.38 (1.11)* ^*b*^	N/A	3.05	−3.80	−2.64
Indecisiveness (0.71)	2.74 (1.08)^a^	2.06 (1.02)^b^	N/A	0.65	−1.20	−0.14
		*2.36 (1.00)* ^*a*^	N/A	0.37	−0.91	0.16
Study 4	*N *=* *59	*N = 63*	*df *=* *120			
Need for more (0.89)	5.34 (0.89)	2.99 (1.24)	11.98***	2.18	1.96	2.74
Making trade‐offs (0.91)	5.76 (0.94)	2.82 (1.08)	16.01***	2.90	2.58	3.30
Indecisiveness (0.76)	2.77 (1.20)	2.59 (1.09)	*ns*	0.16	−0.23	0.59

*Note*

Study 3: Means with different superscripts differ significantly (*p *<* *.05).

Study 3: The means and *SD*s of the “No‐scarcity extra” condition are presented in italics.

N/A = not applicable.

**p <* *.01; ***p <* *.001.

See Supplemental Table 3 for results of scarcity‐related states. No significant differences between conditions were found in engagement, cognitive load, stress, and affect (*p* > .20).

#### Test of Hypotheses: Calories Consumed

Checking the analysis of covariance (ANCOVA) assumption of homogeneity of regression slopes for the full sample (*N *=* *95) revealed a significant interaction between the mean centred covariate hunger and condition, *F*(1, 91) = 5.85, *p *=* *.018, $\eta_{p}^{2}$ = .06. Hence, this assumption was violated and hunger cannot be used as a covariate in an ANCOVA model. To test the hypothesised main effect of condition on calorie consumption after checking all assumptions, a full model ANCOVA with gender as a covariate and condition, hunger, and their interaction on log‐transformed calories consumed was performed. There was no significant main effect of condition on calorie consumption, *F*(1, 90) = 1.08, *p *=* *.30, 95% CI[−0.09, 0.28], $\eta_{p}^{2}$ = .12. Participants in the scarcity condition (*M*_untransformed_ = 126.88, *SD *=* *125.44; *M*_adj, log‐transformed_ = 1.93, *SE *=* *0.06) did not differ in the amount of calories consumed from participants in the no‐scarcity condition (*M*_untransformed_ = 132.68, *SD *=* *148.02; *M*_adj, log‐transformed_ = 1.84, *SE *=* *0.07).

We additionally tested whether the extent of experienced scarcity influenced calorie consumption, irrespective of condition. A multiple regression analysis on “need for more”, “making trade‐offs”, gender, and hunger accounted for 16.5 per cent of the variance in log‐transformed calories consumed, *F*(4, 90) = 4.43, *p *=* *.0003, *R* ^2^ = 16.5. Although the bivariate correlation between “need for more” and log‐transformed calories consumed was marginally significant (*r *=* *.19, *p *=* *.071), “need for more” and “making trade‐offs” did not relate to log‐transformed calorie consumption in the full regression model, β = 0.18, *t*(90) = 1.47, *p *=* *.14, and β = −0.02, *t*(90) = −0.51, *p *=* *.61, respectively.

#### Test of Hypotheses: Desire for Snacks and Inhibition of Eating

Average desire for snacks presented correlated positively to calories consumed, *r *=* *.42 *p *<* *.001, but inhibition of eating was not correlated with calories consumed, *r *=* *−.02, *p *=* *.84. Controlling for gender and hunger, no differences between the scarcity condition (*M*_adj_ = 3.62, *SE *=* *0.13) and the no‐scarcity condition (*M*_adj_ = 3.59, *SE *=* *0.01) were found in desire for snacks, *F*(1, 91) = 0.03, *p *=* *.86, 95% CI[−0.33, 0.39], $\eta_{p}^{2}$ = .00. Neither the scarcity condition (*M *=* *3.58, *SD *=* *1.68) nor the no‐scarcity condition (*M *=* *3.89, *SD *=* *1.40) differed in reported inhibition of eating, *F*(1, 91) = 0.91, *p *=* *.34, 95% CI[−0.94, 0.33], $\eta_{p}^{2}$ = .01.

#### Exploratory Analyses

For exploratory reasons, we further disentangled the non‐hypothesised interaction between hunger and condition that was found upon checking the ANCOVA assumption of homogeneity of regression slopes. Simple slope analyses (see Aiken & West, 1991) demonstrated that for participants with a low level of hunger (−1*SD*), conditions differed on the calories consumed, such that in the scarcity condition significantly more calories were consumed than in the no‐scarcity condition (β = −0.35, *t*(91) = −2.64, *p *=* *.01). However, for participants with a high level of hunger (+1*SD*), no significant difference between conditions was observed (β = 0.10, *t*(91) = 0.78, *p *=* *.44). Checking the assumption of homogeneity of regression slopes without two outliers (two participants consumed a disproportionate amount of calories, *z*‐scores > 4) revealed a non‐significant (or “marginally” significant) interaction between hunger and condition on square‐root transformed calories, *F*(1, 89) = 3.42, *p *=* *.068, $\eta_{p}^{2}$ = .04. Exclusion of the two outliers did not change the results of tests of hypotheses.

Since consuming a large amount of calories may be especially defeating for individuals who have the goal to act otherwise (see also Brownell, 1991; Heatherton et al., 1991), we also tested whether restraint eating goal interacted with scarcity condition on calorie consumption. This interaction was not significant, *F*(5, 82) = 1.04, *p *=* *.40, indicating that the effect of scarcity on the amount of calories consumed did not depend on participants' restraint eating goal.

### Discussion

In Study 2 no support was found for the hypothesised main effect of scarcity on unhealthy food intake, desire for snacks, or inhibition of eating. Although not hypothesised, a (marginally significant) interaction between condition and hunger was found. Scarcity appeared to affect calorie consumption under low hunger levels. Hunger is a strong primary motive that overrules alternative motives (Loewenstein, 1996), and it is plausible that people would be more sensitive to scarcity under situations where such strong biological motives are not active. Hence, our findings concerning the effect of scarcity on eating behaviour remain inconclusive. Furthermore, we noted that control group participants reported a rather high level of scarcity. Even when all options could be chosen, the task may have evoked feelings of wanting to have more. This highlights the theoretical notion that experienced scarcity depends not only on objective resources but also on personal tastes and subjective perception of how much is needed to accomplish (Mullainathan & Shafir, 2014). To test the scarcity hypothesis under more stringent conditions, the experiment was replicated with an improved scarcity manipulation in a sample with low hunger level.

## STUDY 3: REFINING THE SCARCITY MANIPULATION

In Study 3 we aimed to improve the scarcity manipulation used in Study 2. More specifically, by making small changes to the design of the manipulation we aimed to limit experiences of scarcity in participants in the no‐scarcity condition.

### Method

#### Participants, Design, and Procedure

The design and procedure of this study were similar to Study 1 (*N *=* *115, 30 men, mean age 20.27, *SD *=* *1.62, range 17–25). Two changes were made to the trade‐off task compared to Study 2: two new categories were added, and one option was added to each category.^3^ Hence, the trade‐off task consisted of 10 categories of four options. One extra no‐scarcity condition was created in which participants could freely add options to each category (i.e. the “no‐scarcity extra condition”). To limit the induction of extra effort of this no‐scarcity extra condition compared to the no‐scarcity condition, we added a sentence to the instruction that additional options were only to be filled in when there was a desire to add something extra. Another sentence was added to the instruction of all conditions stating that for each category a “restricted budget” (scarcity condition) versus a “certain budget” (no‐scarcity conditions) had been provided by the university. Adding this phrase was done to provide a logical reason—related to financial resources—why participants could choose only one option (scarcity condition) versus multiple options (no‐scarcity) per category. See Supplemental Information for the “no‐scarcity extra” version of the trade‐off task. Participants in one‐third of the lecture room were assigned to the scarcity condition, one‐third of students in the lecture room were assigned to the no‐scarcity condition where all (up to four) options per category could be selected, and the final one‐third was assigned to the no‐scarcity extra condition where all options could be selected plus one idea could be added (five options in total).

### Results

#### Experiences of Scarcity and Indecisiveness

One‐way ANOVAs showed that there was a significant difference between the three conditions on “need for more”, *F*(2, 112) = 50.54, *p *<* *.001, “making trade‐offs”, *F*(2, 112) = 99.16, *p *<* *.001, and “indecisiveness”, *F*(2, 110) = 4.04, *p *=* *.020. Table 2 reports the means, *SD*s per condition on each of these scales, and the post‐hoc results, Cohen's *d* effect sizes, and confidence intervals. Post‐hoc tests (Dunnett's) revealed that a higher need for more and trade‐off making was reported in the scarcity condition compared to both of the no‐scarcity conditions, *p *< .001. Significantly more indecisiveness was reported in the scarcity condition compared to the no‐scarcity condition, *p *=* *.010, but not compared to the no‐scarcity extra condition, *p *=* *.20. See Supplemental Table 4 for results of scarcity‐related states (only engagement and cognitive load were assessed). The scarcity condition scored higher on engagement than the no‐scarcity extra condition (*p *<* *.01) but not compared to the no‐scarcity condition (*p *=* *.46). There was no difference on cognitive load between the scarcity condition and the no‐scarcity conditions (*p *> .09).

### Discussion

Small adjustments in the design of the trade‐off task resulted in an improved scarcity manipulation as the no‐scarcity conditions generally reported lower means (and standard deviations) for need for more and making trade‐offs in this study than in Study 1 and Study 2. As the no‐scarcity extra condition reported the lowest means for need for more and making trade‐offs, this no‐scarcity condition was used in Study 4.

## STUDY 4: TESTING THE EFFECT OF SCARCITY ON UNHEALTHY FOOD INTAKE UNDER LOW LEVEL OF HUNGER

Study 4 was a replication of Study 2 under more stringent conditions. Specifically, in this experiment we explicitly instructed participants to have eaten within 1 hour prior to participation, and used the improved scarcity manipulation of Study 3. We hypothesised that with the improved scarcity manipulation and with a sample with low hunger, scarcity results in more calorie consumption, and a higher desire for snacks and lower inhibition of eating.

### Method

#### Participants and Design

In addition to the participant recruitment strategies used in Study 2, students seated in the university canteen were approached and requested to participate within 1 hour after finishing their meal. A greater sample size than Study 2 was desirable to allow exclusion of participants not adhering to the instruction to eat prior to participation (see Procedure). As no exact estimation of this exclusion could be made, and no preliminary analyses or calculations were performed during the data collection, recruitment efforts were increased over a 3‐week period during which the laboratory rooms were available. The procedure was pre‐tested among three students who were not included in the analysis. One hundred and forty‐one students participated in a two‐group between‐subjects experiment in exchange for a monetary reward of 5 euros. Sixteen participants were excluded from analyses because they did not adhere to the inclusion criterion to eat within 1 hour prior to the experiment (see Procedure). Three participants who had an allergy related to the presented snacks were excluded. Hence, the sample for analysis consisted of 122 participants (20 men), with an average age of 20.26 (*SD *=* *2.10, range 18–31).

#### Procedure and Measures

The procedure was identical to Study 2, except for the following adaptations related to the aim to form a sample with low level of hunger. Participants were scheduled for an individual laboratory session between 08.30 and 11.00 h and between 12.00 and 15.00 h as these times plausibly were closely preceded by breakfast and lunch. Furthermore, participants were instructed verbally (in the university canteen) or by email to have eaten within 1 hour before participation. Upon arrival at the laboratory room, participants were verbally asked whether they had eaten in the last hour. If the answer was no, they were asked to make a new appointment for participation (this occurred four times). One item was added to the pre‐test questionnaire, to check more objectively when was the last time participants had eaten (i.e. “When did you eat *last*?”). As in Study 2, none of the participants identified the true purpose of the study.^4^

### Results

#### Descriptives and Comparability between Conditions

A square‐root transformation on calories consumed was used as this transformation resulted in normally distributed residuals. Participants reported having a goal to eat healthily (*M *=* *5.61, *SD *=* *0.90), and a somewhat restraint eating goal (*M *=* *4.41, *SD *=* *1.34). Participants indicated liking the snacks they consumed (*M*_M&Ms_ = 5.96, *SD *=* *1.17; *M*_popcorn_ = 4.65, *SD *=* *1.61; *M*_crisps_ = 5.38, *SD *=* *1.52; *M*_crispy coated peanuts_ = 4.64, *SD *=* *1.48). This sample reported an average hunger level of 2.16 (*SD *=* *1.11) on a 7‐point rating scale. Conditions did not differ on pre‐test variables age, hunger, healthy eating goal, restraint eating goal, *t*(120) > 0.24, *p* > .14, and gender, *χ* ^*2*^(1, *N* = 122) = 0.11, *p *=* *.74, indicating successful randomisation. As those in the scarcity condition reported experiencing significantly more boredom after the task (*M *=* *3.86, *SD *=* *1.59) compared to the no‐scarcity condition (*M *=* *3.13, *SD *=* *1.61), *t*(120) = 2.54, *p *=* *.012, boredom was included as a covariate in the analyses of calories consumed. Analysis of correlations between the control variables and dependent variables resulted in the identification of gender and hunger as additional covariates in the analyses of calories consumed, age, and hunger as covariates in the analyses of desire for snacks, and age in the analyses of inhibition of eating. Means, *SD*s, and correlations of all variables under study are reported in Supplemental Table 5.

#### Manipulation Check and Exploration of States

*T*‐tests showed that participants in the scarcity condition scored significantly higher than those in the no‐scarcity condition on experienced scarcity scales “need for more” and “making trade‐offs”, but not on “indecisiveness”. See Table 2 for the results of this manipulation check. See Supplemental Table 6 for results of scarcity‐related states. Participants in the scarcity condition scored higher on engagement than those in the no‐scarcity condition (*p *<* *.01). No differences between conditions were found on cognitive load (*p *=* *.44), affect (*p *=* *.42), and stress (*p *=* *.90).

#### Tests of Hypotheses: Calories Consumed^5^

To assess the effect of scarcity condition on calories consumed, an ANCOVA was conducted with gender, hunger, and boredom as covariates. None of the identified covariates (i.e. gender, hunger, boredom) interacted with condition, meaning that the assumption of homogeneity of regression slopes was met. There was no significant main effect of condition on calorie consumption, *F*(1, 117) = 0.02, *p *=* *.88, 95% CI[−1.63, 1.89], $\eta_{p}^{2}$ = .00. Participants in the scarcity condition (*M*_untransformed_ = 130.49, *SD *=* *107.10; *M*_adj, square root‐transformed_ = 10.46, *SE *=* *0.63) did not differ in calories consumed from those in the no‐scarcity condition (*M*_untransformed_ = 132.98, *SD *=* *123.98; *M*_adj, square root‐transformed_ = 10.33, *SE *=* *0.61). Also the extent of experienced scarcity was not related to calories consumed, as “need for more”, β = −0.07, *t*(116) = −0.53, *p *=* *.60, and “making‐trade‐offs”, β = 0.10, *t*(116) = 0.75, *p *=* *.46, were not significant predictors in a multiple regression model including the covariates gender, hunger, and boredom, *F*(5, 116) = 2.20, *p *=* *.06, *R* ^2^ = 0.09.

#### Tests of Hypotheses: Desire for Snacks and Inhibition of Eating

Desire for and inhibition of eating both significantly correlated with calories consumed in the expected direction, respectively *r *=* *.28, *p *=* *.001, and *r *=* *−.27, *p *=* *.003. An ANCOVA controlling for age and hunger showed no differences between the scarcity condition (*M*_adj_ = 3.31, *SE *=* *0.13) and the no‐scarcity condition (*M*_adj_ = 3.48, *SE *=* *0.13) on desire for snacks, *F*(1, 118) = 0.99, *p *=* *.32, 95% CI[−0.53, 0.18], $\eta_{p}^{2}$ = .01. An ANCOVA controlling for age revealed that the scarcity condition (*M*_adj_ = 3.56, *SE *=* *0.21) and no‐scarcity condition (*M*_adj_ = 3.51, *SE *=* *0.20) did not differ in reported inhibition of eating, *F*(1, 119) = 0.03, *p *=* *.86, 95% CI[−0.53, 0.63], $\eta_{p}^{2}$ = .00.

#### Exploratory Analysis

As in Study 2, it was tested whether restraint eating goal interacted with the scarcity condition on calorie consumption. This interaction was again not significant, *F*(5, 109) = 1.18, *p *=* *.33.

### Discussion

In contrast to our expectations, the results of Study 4 indicated that scarcity did not result in more calorie consumption, a higher desire for snacks, or a lower inhibition of eating, in a sample with relatively low self‐reported hunger. The effect sizes of experienced scarcity were greater than those in Study 2, indicating that the manipulation used in Study 4 resulted more successfully in the induction of scarcity versus no scarcity experiences.

## GENERAL DISCUSSION

The present research indicates that although the trade‐off task seemed to evoke scarcity experiences, these do not affect eating behaviour (calorie consumption and self‐regulation of eating). Hence, whereas previous studies showed that scarcity and trade‐off making negatively affect cognitive and attentional outcomes (Mani et al., 2013; Shah et al., 2012), the current research could not support the notion that directly experiencing scarcity also resulted in unhealthy eating. Our results are in line with a longitudinal study of Prentice et al. (2017) that found that at‐the‐moment financial strain was not associated with health behaviours and a diet‐related outcome of overweight. However, there are also studies that show a relation between scarcity and eating behaviour, albeit not experimentally. We discuss three dominant explanations for the inconsistency in the literature regarding this relationship. These explanations may shed light on a more precise conceptualisation and operationalisation of scarcity‐induced eating.

First, it may be that scarcity needs to be experienced as urgent and personally threatening to observe an effect on eating behaviour. This is in line with the suggestion that the enhanced focus on scarcity results particularly from its threat to well‐being: inability to fulfil one's basic needs can have negative and immediate, personal consequences (Koster, Crombez, Van Damme, Verschuere, & De Houwer, 2004; Mullainathan & Shafir, 2013). An experimental study on perceived poverty found that manipulating perceived financial scarcity by reading and writing a text about personal experiences with poor versus rich circumstances did affect subsequent calorie intake (Bratanova et al., 2016). Likewise, a cross‐sectional study found that financial strain, as measured by questions asking to what extent participants could make ends meet and experienced financial difficulties in paying bills for basic needs (e.g. food, electricity) in the preceding year, was associated with decreased fruit and vegetable intake (Beenackers et al., 2017). Also, a longitudinal study showed that evaluations of the family as very poor to just getting by given needs and financial responsibilities increased calorie but decreased fruit and vegetable consumption (Venn & Strazdins, 2017). In contrast to these studies, the present study did not involve personal money resources for meeting basic living needs (poverty concerns), but hypothetical others' resources (i.e. of the university) for meeting needs related to a luxurious event. We reasoned that, following the basic definition of experiencing scarcity (i.e. “a subjective sense of having more needs than resources”; Mullainathan & Shafir, 2014, p. 86), scarcity could be experienced as long as resources are insufficient to fulfil needs and desires. Thereby it was assumed that a certain student culture would shape these needs and desires, allowing us to compose a uniform trade‐off task. Indeed, scarcity was experienced according to these definitions, yet this did not translate to unhealthier eating.

Second, scarcity may have a more pronounced effect on behaviour when it is relative rather than absolute. It has been suggested that subjective experience of scarcity may not be best shaped by absolute availability of resources, but instead by social comparisons with the wealth of others (Festinger, 1954; Sim, Lim, Forde, & Cheon, 2018). Growing evidence shows that subjective perception of own worth compared to others may be more predictive of health than objective, absolute SES indicators including income (Adler, Epel, Castellazzo, & Ickovics, 2000; Boyce, Brown, & Moore, 2010). Based on the proposition that upward social comparisons are a particularly powerful drive for compensation with resources (e.g. food), a recent experimental study showed that personal relative deprivation increases calorie selection and intake (Sim et al., 2018). Plausibly, in the current research scarcity would be experienced to a higher extent when the manipulation involved an upward (versus downward) comparison with others who received more (versus fewer) resources and this provides an interesting direction for future studies.

Third, chronic experiences of scarcity may be more relevant in explaining unhealthy eating than acute or temporal experiences. Longitudinal studies have concluded that persistent, chronic financial scarcity or stress, in particular, results in less healthy eating behaviours (Siahpush et al., 2014; Venn & Strazdins, 2017). The present study aimed to test whether temporarily induced scarcity affects eating behaviour, which is in line with both scarcity theory and self‐regulation theory. For example, a correlational study found that shopping (an economic decision‐making activity) is associated with more simultaneous eating among poorer and not among richer people (Spears, 2011). However, the influence of income scarcity on eating behaviour may come forward in particular when chronic threats to well‐being occur (e.g. savings are drawn). Prolonged experiences of income scarcity may stimulate the development of eating habits that undermine a healthy diet.

Altogether, it can be argued that scarcity needs to be experienced in a sufficiently intrusive way for observing an effect on eating behaviour. Notably, the manipulation in the present study was inspired by previous successful studies showing cognitive effects of dealing with scarce resources, using manipulations involving game playing (Shah et al., 2012) and choosing gifts (Spears, 2011). Although it appeared that we succeeded in manipulating experiences of scarcity, this experience may have been insufficiently intrusive to affect a multifactorially determined behaviour such as food intake. This was also reflected by the inconsistent results of the scarcity‐related processes that were assessed in each of the four studies and that could act as mechanisms in the effect of scarcity on snack intake (an effect on mental engagement was observed in three studies; on cognitive load only in the first study). However, this conclusion can only be drawn tentatively given the psychometric quality of the measures (i.e. with the exception of engagement, the process variables were measured with one item). Null findings may also be due to a lack of power; however, since our sample size is larger than was predetermined in a power calculation and exceeds those in previous similar experiments including food consumption (Bratanova et al., 2016; Sim et al., 2018), a lack of power does not seem a satisfactory explanation.

Although not a priori hypothesised, in the first laboratory experiment (Study 2), an interaction between the scarcity condition and hunger bordered on significance, such that scarcity may only affect calorie consumption under low levels of hunger. This finding was intriguing as a similar pattern was observed in a correlational study by Hill, Prokosch, DelPriore, Griskevicius, and Kramer (2016): participants raised in low socioeconomic status (SES) neighbourhoods, characterised by scarcity of resources, consumed a high amount of calories independent of their energy need, whereas those raised in high SES neighbourhoods regulated their caloric consumption according to their energy need. Based on Life History theory, the authors suggested that growing up in resource‐scarce environments stimulates eating in the absence of hunger as this would promote survival. Bratanova et al. (2016) also suggested that the effect of poverty perceptions on food intake would occur in the absence of hunger, although this was not tested in their studies. However, in our second laboratory experiment (Study 4) in which participants were explicitly instructed to eat prior to participation, an effect of scarcity under low hunger level could not be replicated. Altogether, we conclude that no effect of scarcity on eating was found. We advise future research on the relationship between scarcity and eating behaviour to assess or manipulate hunger level.

### Limitations and Strengths

The present study also has limitations that need to be acknowledged in these interpretations. First, scarcity‐related processes (e.g. cognitive load, stress) and indications of self‐regulation of eating were mainly measured by single‐item retrospective measures, which may not have been reliable. Although calorie consumption was the main focus of the study, it would be an interesting direction for future research studying the effect of scarcity on eating to check a scarcity manipulation not only by measuring perceived scarcity but also to assess these processes more thoroughly. For instance, state cognitive control can be more directly assessed with a computer task measuring impulse inhibition and stress more objectively by blood pressure and heart rate measures. Second, we did not measure participants' own income level and financial strain in their personal life, and hence we cannot rule out that personal scarcity was not equal between conditions. However, we did not expect an influence of income for two reasons. First, the laboratory experiments were based on random assignment. Therefore, one would not expect differences in income between conditions. Second, in our manipulation participants were (hypothetically) *put in a new situation* of trying to fulfil needs under scarcity (versus no‐scarcity); this would induce a direct experience of scarcity. It is implausible that participants would think of their personal financial situation when conducting this task. Third, as we did not assess time to complete the trade‐off task in the laboratory studies, it remains unclear whether differences in duration of completion between conditions may have differentially affected cognitive capacity and eating. Fourth, we only assessed immediate calorie intake of snacks as eating such tasty but unhealthy foods was considered particularly susceptible to impulsive tendencies, but it would also be of interest to test whether scarcity affects other eating behaviours that contribute to unhealthy eating patterns, including food choice, consumption of main meals, and overall daily calorie intake. Nevertheless, this is one of the first studies to experimentally investigate the direct causal effect of scarcity experiences on (one type of) eating behaviour. Future studies may investigate more lasting effects of scarcity on eating behaviours that contribute to unhealthy eating patterns. Our study was distinct from previous studies in that it focused on acute dealing with scarcity, involving trade‐offs and sacrifices that reinforce the feeling of having less than needed (Mullainathan & Shafir, 2013). This study succeeded in developing a successful trade‐off task including various needed and desirable options, resulting in experiences of having too little and wanting more. To our knowledge, this study was the first to comprehensively check whether objective forms of scarcity (in this study receiving few resources to choose options) translate to subjective experiences of scarcity while these are in essence shaped by personal evaluations.

### Conclusion

In conclusion, our studies did not show an acute effect of experienced scarcity on caloric intake. We argue that not all forms of experienced scarcity are sufficiently threatening to affect eating behaviours. Rather, based on previous successful studies, we suggest that scarcity posing a threat to personal well‐being, a relative form of scarcity, or a more persistent experience of scarcity may be more likely to have negative consequences for healthy eating. Our findings call for a more nuanced view of scarcity and how and under what circumstances scarcity affects eating behaviour. Future research should sharpen the conceptualisation of scarcity and evaluate specific elements of scarcity in their relevance to eating behaviour. These insights could inform new psychological interventions for decreasing diet quality disparities between income groups.

## CONFLICT OF INTEREST

The authors have no conflict of interest to declare.

## Supporting information


**Study 1**– Measurements of states related to scarcity
**Supplemental Table 1.** Results of t‐tests comparing the two conditions on scarcity related states in Study 1
**Supplemental Table 2.** Means, SDs, and Correlations of the Variables under Study in Study 2 (N = 95)
**Supplemental Table 3.** Results of t‐tests comparing the two conditions on scarcity related states in Study 2
**Supplemental Table 4.** Post hoc results (Dunnett's tests) comparing both no‐scarcity conditions with the scarcity condition on scarcity related states in Study 3
**Supplemental Table 5.** Means, SDs, and Correlations of the Variables under Study in Study 4 (*N* = 122)
**Supplemental Table 6.** Results of t‐tests comparing the two conditions on scarcity related states in Study 4
Trade‐off task (scarcity manipulation) as used in Study 1, presented is the scarcity condition, translated from Dutch to English
Trade‐off task (scarcity manipulation) as used in Study 2, presented is the scarcity condition, translated from Dutch to English
Trade‐off task (scarcity manipulation) as used in Study 3 and 4, presented is the “no‐scarcity extra” condition, translated from Dutch to English

## Notes

1 The construction of the list of categories with desirable, competing options was based on a discussion with three fellow researchers and a pre‐test among 30 students, respectively. From a list of 13 categories, students were asked to choose one option per category, and to mark five categories they perceived as the most difficult trade‐offs. The nine most marked categories were included in Study 1.

2 Excluding 11 participants who mentioned snack intake as a possible purpose did not change the pattern of results.

3 The added options were based on frequently reported answers on a filler question in the post‐test questionnaire in Study 2, asking what items participants would add for an ideal party.

4 Excluding 17 participants who mentioned snack intake did not change the pattern of results.

5 The tests of hypotheses were also performed on a subsample of participants reporting little hunger. Excluding 16 participants from the current sample who rated their hunger level as more than 3 on a 7‐point rating scale (i.e. 4, 5, 6, or 7) did not affect the results of the tests of hypotheses as reported.
