# Supplementary material for: Dealing with Too Little: The Direct Experience of Scarcity does not Affect Snack Intake
Source: Appl Psychol Health Well Being. 2019 Apr 9;11(3):459–83. doi: 10.1111/aphw.12163 (PMC6899634; doi:10.1111/aphw.12163)
Supplement: Supplementary file 1 — Study 1– Measurements of states related to scarcity Supplemental Table 1. Results of t‐tests comparing the two conditions on scarcity related states in Study 1 Supplemental Table 2. Means, SDs, and Correlations of the Variables under Study in Study 2 (N = 95) Supplemental Table 3. Results of t‐tests comparing the two conditions on scarcity related states in Study 2 Supplemental Table 4. Post hoc results (Dunnett's tests) comparing both no‐scarcity conditions with the scarcity condition on scarcity related states in Study 3 Supplemental Table 5. Means, SDs, and Correlations of the Variables under Study in Study 4 (N = 122) Supplemental Table 6. Results of t‐tests comparing the two conditions on scarcity related states in Study 4 Trade‐off task (scarcity manipulation) as used in Study 1, presented is the scarcity condition, translated from Dutch to English Trade‐off task (scarcity manipulation) as used in Study 2, presented is the scarcity condition, translated from Dutch to English Trade‐off task (scarcity manipulation) as used in Study 3 and 4, presented is the “no‐scarcity extra” condition, translated from Dutch to English [file APHW-11-459-s001.docx]

**Supporting information**

**Study 1 – Measurements of states related to scarcity**

Cognitive engagement in making choices was assessed with a three item measure inspired by Vohs et al. (2014), (e.g. “I was thinking actively while making choices”). Items were answered on a 7-point Likert scale measuring the degree of agreement, ranging from 1 (strongly disagree) to 7 (strongly agree). The scale was reliable, Cronbach’s α = .82 and a mean score was computed. Self-reported cognitive load was measured with one item developed by Paas (1992); Paas and Van Merriënboer (1994) that was validated against different levels of task complexity in the domains of statistics and geometrics (Paas, Van Merriënboer, & Adam, 1994). Participants were asked to rank on a seven-point Likert scale “How much effort did you put in this task?”, ranging from 1 (very low effort) to 7 (very high effort). Affect was with assessed with one item “How do you feel at this moment?”. The answer scale ranged from 1 (very negative) to 7 (very positive).

SUPPLEMENTAL TABLE 1

Results of t-tests comparing the two conditions on scarcity related states in Study 1

| Scale (range 1-7) | Scarcity condition  (*N* = 41) | No-scarcity condition  (*N* = 40) | *t*-test  (*df* = 79) | Cohen's *d* |
| --- | --- | --- | --- | --- |
| Engagement | 5.23 (0.94) | 4.67 (1.20) | 2.37* | .53 |
| Cognitive load | 4.51 (1.10) | 3.85 (1.41) | 2.37* | .52 |
| Affect^a^ | 4.85 (1.11) | 5.10 (1.24) | -0.95 | .21 |

*Note:* * *p* < .05

SUPPLEMENTAL TABLE 3
Results of t-tests comparing the two conditions on scarcity related states in Study 2

| Scale (range 1-7) | Scarcity condition  (*N* = 49) | No-scarcity condition  (*N* = 46) | *t*-test  (*df* = 93) | Cohen's *d* |
| --- | --- | --- | --- | --- |
| Engagement | 5.67 (0.85) | 5.59 (0.79) | *ns* | .10 |
| Cognitive load | 4.57 (1.34) | 4.20 (1.51) | *ns* | .26 |
| Affect | 5.80 (0.82) | 5.61 (0.80) | *ns* | .23 |
| Stress^a^ | 2.10 (1.23) | 1.98 (1.00) | *ns* | .11 |

*Note:* ** *p* < .01. ^a^ assessed with “How stressed were you during the task?”.

SUPPLEMENTAL TABLE 4
Post hoc results (Dunnett's tests) comparing both no-scarcity conditions with the scarcity
condition on scarcity related states in Study 3

|  | Conditions | |  |  |  |
| --- | --- | --- | --- | --- | --- |
| Scale (range 1-7) | Scarcity  (*N* = 39) | No-scarcity  (*N* = 38) | No-scarcity extra (*N* = 38) | Cohen's *d* of scarcity vs no-scarcity | Cohen's *d* of scarcity vs no-scarcity extra |
| Engagement | 5.14 (.79)^a^ | 4.86 (1.06)^a^ | 4.39 (1.40)^b^ | 0.15 | 0.66 |
| Cognitive load | 4.36 (1.33)^a^ | 4.24 (1.40)^a^ | 3.71 (1.59)^a^ | 0.09 | 0.44 |

*Note*: Means with different superscripts differ significantly (*p* < .05)

SUPPLEMENTAL TABLE 6
Results of t-tests comparing the two conditions on scarcity related states in Study 4

| Scale (range 1-7) | Scarcity condition (*N* = 59) | No-scarcity condition (*N* = 63) | *t*-test (*df* = 120) | Cohen's *d* |
| --- | --- | --- | --- | --- |
| Engagement in choosing | 5.84 (0.76) | 5.48 (0.67) | 2.79** | .50 |
| Cognitive load | 4.64 (1.52) | 4.44 (1.34) | 0.77 | .14 |
| Affect^a^ | 5.78 (0.81) | 5.67 (0.74) | 0.80 | .14 |
| Stress^a^ | 2.19 (1.29) | 2.16 (1.13) | 0.13 | .02 |

*Note:* ** *p* < .01

SUPPLEMENTAL TABLE 2
 Means, SDs, and Correlations of the Variables under Study in Study 2 (N = 95)

|  | 1 | 2 | 3 | 4 | 5 | 6 | 7 | 8 | 9 | 10 | 11 | 12 | 13 | 14 | 15 | 16 | 17 | 18 |
| --- | --- | --- | --- | --- | --- | --- | --- | --- | --- | --- | --- | --- | --- | --- | --- | --- | --- | --- |
| 1. Age | - |  |  |  |  |  |  |  |  |  |  |  |  |  |  |  |  |  |
| 2. Gender (% males)^b^ | .04 | - |  |  |  |  |  |  |  |  |  |  |  |  |  |  |  |  |
| 3. Hunger^a^ | .02 | .16 | - |  |  |  |  |  |  |  |  |  |  |  |  |  |  |  |
| 4. Healthy eating goal^a^ | -.08 | .13 | .02 | - |  |  |  |  |  |  |  |  |  |  |  |  |  |  |
| 5. Restraint eating goal^a^ | -.06 | -.01 | -.02 | .30** | - |  |  |  |  |  |  |  |  |  |  |  |  |  |
| 6. Need for more^a^ | .10 | .10 | .08 | -.20 | .00 | - |  |  |  |  |  |  |  |  |  |  |  |  |
| 7. Making trade-offs^a^ | .02 | .05 | .02 | -.07 | -.13 | .62** | - |  |  |  |  |  |  |  |  |  |  |  |
| 8. Engagement^a^ | -.14 | .15 | -.07 | .12 | .02 | -.03 | .12 | - |  |  |  |  |  |  |  |  |  |  |
| 9. Indecisiveness^a^ | -.07 | -.06 | .13 | -.08 | .05 | .20 | -.49** | .11 | - |  |  |  |  |  |  |  |  |  |
| 10. Cognitive load^a^ | .05 | .13 | .03 | .15 | -.10 | .11 | .24* | .42** | .25* | - |  |  |  |  |  |  |  |  |
| 11. Affect^a^ | -.03 | .08 | -.01 | .01 | -.05 | .01 | .04 | .27** | -.21 | .05 | - |  |  |  |  |  |  |  |
| 12. Stress^a^ | -.02 | -.03 | .04 | .03 | .03 | .13 | .17 | -.05 | .36** | .16 | -.29 | - |  |  |  |  |  |  |
| 13. Boredom^a^ | .13 | -.06 | -.03 | -.08 | .10 | .19 | .10 | -.03 | -.01 | .01 | -.01 | -.01 | - |  |  |  |  |  |
| 14. Liking of consumed snacks^a^ | -.12 | .02 | .02 | -.04 | -.07 | -.08 | .07 | .23* | .07 | .08 | .34** | -.13 | .00 | - |  |  |  |  |
| 15. Desire for snacks^a^ | -.08 | .24* | .35** | .10 | -.17 | .08 | .08 | .10 | -.09 | .10 | .03 | .11 | -.10 | .07 | - |  |  |  |
| 16. Inhibition of eating^a^ | -.05 | .13 | .11 | -.14 | -.11 | -.06 | -0.14 | -.06 | .05 | .02 | .00 | .08 | .00 | .19 | .24* | - |  |  |
| 17. Calories consumed | .18 | .27** | .27** | .14 | -.04 | .21* | .03 | .17 | -.14 | .09 | .06 | .04 | .07 | -.11 | .30** | -.07 | - |  |
| 18. Log calories consumed | .11 | .27** | .29** | .04 | -.13 | .19^Ϯ^ | .07 | .06 | -.10 | -.03 | .10 | .13 | -.06 | -.16 | .42** | -.02 | .78** | - |
| *Mean* | 20.83 | 12.6 | 3.37 | 5.49 | 4.40 | 4.75 | 4.22 | 5.63 | 2.50 | 4.39 | 5.71 | 2.04 | 3.68 | 5.47 | 14.41 | 3.73 | 129.69 | 1.90 |
| *SD* | 2.20 |  | 1.67 | 1.01 | 1.28 | 1.39 | 1.78 | .82 | 1.04 | 1.43 | .81 | 1.12 | 1.55 | 1.06 | 3.73 | 1.55 | 136.18 | .48 |

*Note*: * correlation significant at *p* < .05, ** correlation significant at *p* < .01, ^Ϯ^ correlation marginally significant at *p* = .071. ^a^ measured by a 7-point Likert scale ranging from 1-7. ^b^ Spearman’s rho correlation (all other are Pearson correlation coefficient.

SUPPLEMENTAL TABLE 5
Means, SDs, and Correlations of the Variables under Study in Study 4 (*N* = 122)

|  | 1 | 2 | 3 | 4 | 5 | 6 | 7 | 8 | 9 | 10 | 11 | 12 | 13 | 14 | 15 | 16 | 17 | 18 |
| --- | --- | --- | --- | --- | --- | --- | --- | --- | --- | --- | --- | --- | --- | --- | --- | --- | --- | --- |
| 1. Age | - |  |  |  |  |  |  |  |  |  |  |  |  |  |  |  |  |  |
| 2. Gender (% males)^b^ | -.11 | - |  |  |  |  |  |  |  |  |  |  |  |  |  |  |  |  |
| 3. Hunger^a^ | -.03 | -.22* | - |  |  |  |  |  |  |  |  |  |  |  |  |  |  |  |
| 4. Healthy eating goal^a^ | .08 | .20* | -.25** | - |  |  |  |  |  |  |  |  |  |  |  |  |  |  |
| 5. Restraint eating goal^a^ | .15 | .24** | -.22* | .23* | - |  |  |  |  |  |  |  |  |  |  |  |  |  |
| 6. Need for more^a^ | -.06 | -.08 | -.00 | -.01 | .19* | - |  |  |  |  |  |  |  |  |  |  |  |  |
| 7. Making trade-offs^a^ | -.07 | -.04 | .01 | -.04 | .06 | .72** | - |  |  |  |  |  |  |  |  |  |  |  |
| 8. Engagement^a^ | -.07 | -.04 | -.14 | -.12 | .04 | .16 | .25** | - |  |  |  |  |  |  |  |  |  |  |
| 9. Indecisiveness^a^ | .07 | -.09 | .05 | -.22* | -.04 | .12 | .37** | .07 | - |  |  |  |  |  |  |  |  |  |
| 10. Cognitive load^a^ | .02 | .04 | -.10 | -.03 | -.18* | .06 | .15 | .29** | .19* | - |  |  |  |  |  |  |  |  |
| 11. Affect^a^ | .15 | .03 | -.23* | .15 | .06 | .12 | .08 | .18* | -.14 | .07 | - |  |  |  |  |  |  |  |
| 12. Stress^a^ | .01 | .03 | -.06 | -.03 | -.04 | .06 | .13 | .15 | .33** | .24** | -.21* | - |  |  |  |  |  |  |
| 13. Boredom^a^ | -.03 | -.14 | .09 | .13 | .13 | .25** | .22* | -.06 | -.08 | -.07 | .08 | .02 | - |  |  |  |  |  |
| 14. Liking of consumed snacks^a^ | -.04 | .21* | -.19* | .06 | .05 | -.03 | .01 | .15 | .03 | .13 | .23* | -.01 | .10 | - |  |  |  |  |
| 15. Desire for snacks^a^ | -.21* | -.09 | .19* | -.07 | -.06 | -.01 | .02 | .18* | -.00 | .06 | -.01 | .01 | -.03 | .15 | - |  |  |  |
| 16. Inhibition of eating^a^ | -.19* | .07 | -.03 | .03 | .19** | .12 | .10 | .03 | .05 | .03 | -.18 | .05 | .01 | .09 | .24** | - |  |  |
| 17. Calories consumed | -.03 | -.21* | .22* | .01 | -.09 | -.01 | .03 | .09 | -.09 | .08 | .15 | .03 | .07 | .02 | .29** | -.27** | - |  |
| 18. Square root calories | -.03 | -.21* | .22* | -.01 | -.09 | .03 | .06 | .09 | -.07 | .09 | .17^Ϯ^ | .02 | .10 | .07 | .32** | -.24** | .97** | - |
| *Mean* | 20.26 | 16.4 | 2.16 | 5.61 | 4.41 | 4.13 | 4.24 | 5.66 | 2.68 | 4.54 | 5.72 | 2.17 | 3.48 | 5.53 | 3.40 | 3.53 | 131.78 | 10.39 |
| *SD* | 2.10 |  | 1.11 | .90 | 1.34 | 1.60 | 1.79 | .73 | 1.14 | 1.43 | .77 | 1.20 | 1.17 | 1.07 | 1.02 | 1.63 | 115.48 | 4.90 |

*Note*: * correlation significant at *p* < .05, ** correlation significant at *p* < .01.
 ^a^ measured by a 7-point Likert scale ranging from 1-7. ^b^ Spearman’s rho correlation (all other are Pearson correlation coefficient).

**Trade-off task (scarcity manipulation) as used in Study 1, presented is the scarcity condition, translated from Dutch to English**

**Introduction**Because of an anniversary of your study, Wageningen University wants to give a party for all students of your study. The party will be held in the café of the Forum. The area is empty and available from 21.00 until 03.00 hours.
Imagine that *you* are chosen to organise this party.

**Instruction**Your task is to choose which goods and services need to be ordered and arranged. Try to put yourself in the role of the organisation. Imagine you want this party to be a success. Your choices are important, because these determine the content of the party. To thank you for your input, your name will be written on the invitation.

Below you will see 9 categories of goods and services that may be important for the party. Every category consists of 3 different options. You may assume all options within a category are equally expensive.

From the following rows of 3 options, **you may choose only 1 option (by ticking the box) that you want for the party.**

1. Personnel  *Which personnel do you want to hire? (Maximum 1 option possible)*□ DJ

□ Toilet personnel

□ Bar personnel

2. Promotion
*Which promotion option do you want? (Maximum 1 option possible)*□ E-mail

□ Facebook

□ Posters/flyers

3. Facilities 1
*Which facility do you want to arrange? (Maximum 1 option possible)*□ Standing tables

□ Dance floor

□ Lounge corner / seating area

4. Facilities 2
*Which facility do you want to arrange? (Maximum 1 option possible)*
□ Bicycle shed

□ Cloakroom

□ Smoking area

5. Drinks *Which drink do you want to arrange? (Maximum 1 option possible)*
□ Beer

□ Wine

□ Soda

6. Music to be played
*Which music do you want to be played? (Maximum 1 option possible)*□ 90’s hits

□ Top 40

□ Dance

7. Times of playing music
*At which time do you want to play music? (Maximum 1 option possible)*□ 21.00-23.00 hours

□ 23.00-01.00 hours

□ 01.00-03.00 hours

8. Part of karaoke activity  *Which part of the karaoke activity do you want to arrange? (Maximum 1 option possible)*□ Microphone

□ Beamer with screen for the lyrics

□ CD with music in karaoke verison muziek (instrumental)

9. Further items
*Which further item do you want to arrange? (Maximum 1 option possible)*□ Glasses

□ Beer tap

□ Music (CDs)

**Trade-off task (scarcity manipulation) as used in Study 2, presented is the scarcity condition, translated from Dutch to English**

**Introduction**Wageningen University exists for 100 years in 2018. Let’s party! How would you organize the party? Please think along with us.

The party will be held in an area with a bar and toilets, but for the rest the area will be empty . The area is available from 21.00 until 03.00 hours.

Imagine that *you* are chosen to organise this party for your fellow students.

**Instruction**Your task is to choose which goods and services need to be ordered and arranged. The university has already composed a list with categories and options. Try to put yourself in the role of the organisation. Imagine you want this party to be a great success. Your choices are important, because these determine the content of the party.

Below you will see 8 categories of goods and services that may be important for the party. Every category consists of 3 different options. You may assume all options within a category are equally expensive.

From the following rows of 3 options, **you may choose only 1 option (by ticking the box) that you want for the party.**

1. Personnel  *Which personnel do you want to hire? (Maximum 1 option possible)*□ DJ

□ Photographer

□ Bar personnel

2. Promotion
*Which promotion option do you want? (Maximum 1 option possible)*□ E-mail

□ Facebook

□ Posters/flyers

3. Facilities 1
*Which facility do you want to arrange? (Maximum 1 option possible)*□ Standing tables

□ Dance floor

□ Lounge corner / seating area

4. Drinks *Which drink do you want to arrange? (Maximum 1 option possible)*
□ Beer

□ Wine and liquor

□ Soda

6. Music to be played
*Which music do you want to be played? (Maximum 1 option possible)*□ 90’s hits

□ Top 40

□ Dance / techno

7. Times of playing music
*At which time do you want to play music? (Maximum 1 option possible)*□ 21.00-23.00 hours

□ 23.00-01.00 hours

□ 01.00-03.00 hours

8. Part of karaoke activity  *Which part of the karaoke activity do you want to arrange? (Maximum 1 option possible)*□ Microphone

□ Beamer with screen for the lyrics

□ CD with music in karaoke verison muziek (instrumental)


9. Bar items
*Which items do you want to at the bar? (Maximum 1 option possible)*□ Glasses

□ Beer tap

□ Glass rinser

**Trade-off task (scarcity manipulation) as used in Study 3 and 4, presented is the ‘no-scarcity extra’ condition, translated from Dutch to English**

**Introduction**Wageningen University exists for 100 years in 2018. Let’s party! How would you organize the party? Please think along with us.

The party will be held in an area with a bar and toilets, but for the rest the area will be empty The area is available from 21.00 until 03.00 hours.

Imagine that *you* are chosen to organise this party for your fellow students.

**Instruction**Your task is to choose which goods and services need to be ordered and arranged. The university has already composed a list with categories and options. Try to put yourself in the role of the organisation. Imagine you want this party to be a great success. Your choices are important, because these determine the content of the party.

Below you will see 10 categories of goods and services that may be important for the party. Every category consists of 3 different options. You may assume all options within a category are equally expensive. Each category based on a certain budget, but there is room for an extra option.

For each category, **you may choose all options (by ticking the box) that you want for the party. The last option is still open, please only fill this in if you readily want to choose something extra.**

1. Personnel  *Which personnel do you want to hire? (More options possible)*□ DJ

□ Toilet personnel

□ Bar personnel

□ Doorkeeper

□ .........................................

2. Promotion
*Which promotion option do you want? (More options possible)*□ E-mail

□ Facebook

□ Posters/Flyers

□ During lectures

□ .........................................

3. Facilities
*Which facility do you want to arrange? (More options possible)*□ Standing tables

□ Dance floor

□ Lounge corner/Seating area

□ Wardrobe

□ .........................................

4. Drinks *Which drink do you want to arrange? (More options possible)*
□ Beer

□ Wine

□ Soda

□ Cocktails

□ .........................................

5. Music to be played
*Which music do you want to be played? (More options possible)*□ 80’s/90’s hits

□ Top 40

□ Dance / Techno

□ Rock

□ .........................................

6. Times of playing music
*At which time do you want to play music? (More options possible)*□ 21.00-23.00 hours

□ 23.00-01.00 hours

□ 01.00-03.00 hours

7. Part of karaoke activity  *Which part of the karaoke activity do you want to arrange? (More options possible)*□ Microphone

□ Beamer with screen for the lyrics

□ CD with music in karaoke version music (instrumental)

□ Stage

□ .........................................

8. Event

In addition to karaoke, (an) event(s) can be arranged. Which event(s) do you want to arrange? (*More options possible*)

□ Joint activity (e.g. games, dance workshop)

□ Speech (for example, by the head of the university)

□ Live band

□ Happy hour

□ .........................................

9. Items for the bar
*Which item for the bar do you want to arrange? (More options possible)*□ Drinking glasses

□ Refrigerators

□ Beer tap

□ Rinsing tube

□ .........................................

10. Stage setting

Which stage setting do you want to arrange? *(More options possible)*

□ Decoration

□ Illumination and disco lights

□ Snacks

□ Welcome drink

□ .........................................

**References**

Paas, F. G. W. C. (1992). Training strategies for attaining transfer of problem-solving skill in statistics: A cognitive-load approach. *Journal of Educational Psychology, 84*(4), 429-434.

Paas, F. G. W. C., & Van Merriënboer, J. J. G. (1994). Variability of worked examples and transfer of geometrical problem-solving skills: A cognitive-load approach. *Journal of Educational Psychology, 86*(1), 122-133.

Paas, F. G. W. C., Van Merriënboer, J. J. G., & Adam, J. J. (1994). Measurement of cognitive load in instructional research. *Perceptual and Motor Skills, 79*(1), 419-430.

Vohs, K. D., Baumeister, R. F., Schmeichel, B. J., Twenge, J. M., Nelson, N. M., & Tice, D. M. (2014). Making choices impairs subsequent self-control: a limited-resource account of decision making, self-regulation, and active initiative. *Motivation Science, 1*(S), 19-42.
